# Changes in the Morphology and Antioxidant Status of European Red Deer Sperm Stored in the Epididymides and in a Liquid State

**DOI:** 10.3390/ani14111653

**Published:** 2024-05-31

**Authors:** Nicoletta M. Neuman, Aleksandra Orzołek, Żaneta Steiner-Bogdaszewska, Anna Dziekońska

**Affiliations:** 1 Department of Animal Biochemistry and Biotechnology, University of Warmia and Mazury in Olsztyn, Oczapowskiego 5, 10-719 Olsztyn, Poland; nicoletta.neuman@uwm.edu.pl (N.M.N.); aleksandra.deszczka@uwm.edu.pl (A.O.); 2 Witold Stefański Institute of Parasitology of the Polish Academy of Sciences, Research Station in Kosewo Górne, 11-700 Mrągowo, Poland; kosewopan@kosewopan.pl

**Keywords:** epididymal sperm, morphology, antioxidant status, storage, European red deer

## Abstract

**Simple Summary:**

The choice of the optimal sperm preservation method is an important consideration in animal breeding. Stored semen can be used for reproductive purposes to introduce new genotypes and prevent inbreeding, which poses a considerable problem in cervid farms. The aim of this study was to evaluate and compare the effect of storage time and storage method (liquid state/epididymides) on the motility, morphology, and antioxidant status of European red deer sperm stored at 5 °C for up to six days (D0-D6). Sperm samples were assessed for motility, viability, morphology, activity of antioxidant enzymes (superoxide dismutase, SOD; glutathione peroxidase, GPx; catalase, CAT), and lipid peroxidation (based on malondialdehyde, MDA, content). Significant differences between storage variants were noted on D2 in sperm morphology; on D4 in the percentage of progressively motile sperm, MDA content, and SOD and GPx activity; and on D6 in the percentage of motile and viable spermatozoa. Sperm motility, viability, and antioxidant status are more effectively preserved during liquid storage than epididymal storage. Morphological and functional abnormalities of sperm were observed earlier during epididymal storage, which suggests that spermatozoa can be stored for shorter periods of time in the epididymides than in a liquid state.

**Abstract:**

The aim of this study was to evaluate the motility, morphology, and antioxidant status of European red deer sperm stored in a liquid state (variant I) and in the epididymides (variant II). Spermatozoa were harvested post-mortem from the cauda epididymis. Sperm samples in both variants were stored for up to six days (D6) at 5 °C. Spermatozoa were assessed for motility, viability, morphology, activity of antioxidant enzymes (superoxide dismutase, SOD; glutathione peroxidase, GPx; catalase, CAT), and lipid peroxidation (malondialdehyde, MDA, content). Sperm samples were analyzed on storage days 0, 2, 4, and 6 (D0-D6). Storage time and storage method significantly (*p* ≤ 0.05) influenced the examined variables. On D2, a decrease in motility and acrosomal integrity was observed in both storage variants, whereas a decrease in viability and an increase in MDA content were noted in spermatozoa stored in the epididymides. On D4, higher values of SOD and GPx activity and MDA content were noted in variant I than in variant II. Catalase activity was very low. GPx is the key enzyme that participates in the reduction of hydrogen peroxide in sperm cells. Spermatozoa stored in a liquid state were characterized by higher motility and viability, improved morphology and antioxidant status than those stored in the epididymides; therefore, liquid storage is more recommended for short-term preservation of epididymal spermatozoa.

## 1. Introduction

The knowledge about the semen of free-living animals and effective semen preservation methods is limited and should be expanded. The existing sperm storage methods have been developed based on our knowledge of livestock species. Deer sperm is preserved mostly with the use of methods designed for domesticated ruminants [1], and ejaculated semen is recommended for preservation. However, semen samples are difficult to obtain from cervids due to their unique behavior, and sperm is often collected post-mortem from the epididymides [2,3]. The epididymal sperm of cervids, similarly to ejaculated sperm, can be stored in a liquid state [4,5,6], in a frozen state [7,8], and in the epididymides [9,10]. Of these methods, cryopreservation is the most commonly used. However, it is not always recommended for economic and practical reasons. Moreover, the fertilizing capacity of cryopreserved sperm is reduced, compared with fresh sperm and sperm stored for a short period of time in a liquid state [1]. For this reason, short-term sperm storage may be a better method to protect sperm quality than cryopreservation. Epididymal sperm harvested from deer in their natural environment is a source of valuable genetic material for reproductive purposes [11,12]. Their use in assisted reproduction techniques can reduce the phenomenon of inbreeding in limited-population deer farms and contribute to significant breeding progress [2]. However, spermatozoa cannot always be collected from hunter-harvested animals directly after culling, and in some cases, sperm is stored in the epididymides for many hours. Previous studies have shown that prolonged storage in the epididymides can adversely affect sperm functions and their suitability for reproduction [9,13].

The quality of stored spermatozoa can be undermined by various factors, including oxidative stress. Oxidative stress increases lipid peroxidation and disrupts the activity of antioxidant enzymes [14,15]. Oxidative stress also causes oxidative damage to cell organelles and increases the production of reactive oxygen species (ROS) [14,16,17]. Spermatozoa stored at low temperatures are exposed to cold shock. Cold shock enhances ROS production in sperm cells and increases their susceptibility to lipid peroxidation [18].

In healthy organisms, antioxidant defense systems offer protection against the harmful effects of ROS [19,20]. The key antioxidant enzymes include superoxide dismutase (SOD), glutathione peroxidase (GPx), and catalase (CAT) [16,17,19,21]. Sperm cells rely on the antioxidant enzymes in the epididymides and their own antioxidant capacity to mitigate the harmful consequences of ROS during transport through the epididymides, storage, and fertilization [22].

Increased lipid peroxidation and impaired antioxidant status during storage can affect sperm viability, morphology, and motility [23,24]. Sperm morphology is an important consideration during sperm assessment [25]. Spermatozoa with a normal morphology have a higher fertilization potential [26]. Morphological defects, in particular, head defects, significantly undermine sperm’s ability to fuse with an egg cell [25]. Changes in the morphology and antioxidant status of stored cervid epididymal spermatozoa have not been analyzed to date. 

The aim of this study was to evaluate the influence effect of storage time on the motility, morphology, and antioxidant status of spermatozoa stored in a liquid state and in the epididymides at a temperature of 5 °C. Furthermore, the effect of the storage variant (liquid storage vs. storage in the epididymides) on sperm parameters was determined on subsequent days of storage (D2, D4, D6). Sperm motility, viability, sperm morphology (including morphological defects), the activity of antioxidant enzymes (SOD, GPx, CAT), and lipid peroxidation (based on malondialdehyde, MDA, content) were assessed in spermatozoa stored for up to six days. 

## 2. Materials and Methods

### 2.1. Collection of Epididymal Sperm

Testicles and epididymides stored in scrotal sacs were collected from 32 European red deer (*Cervus elaphus elaphus*) stags that were culled during legal hunts in the breeding season (September–October). European red deer were hunter-harvested in the Nowe Ramuki Forest District (Warmian-Masurian Voivodeship, Poland) according to hunting and wildlife management rules. Testicles and epididymides were collected within 5 h after the hunt and were transported to the laboratory of the Department of Animal Biochemistry and Biotechnology of the University of Warmia and Mazury in Olsztyn. 

Sperm samples for analyses were obtained from the cauda epididymis by multiple incisions made with a scalpel and were collected in Eppendorf tubes [5]. During a preliminary assessment of sperm samples, sperm concentrations were determined using a Bürker chamber (Equimed-Medical Instruments, Cracow, Poland). In each sample, sperm motility was assessed subjectively under a microscope [5]. 

### 2.2. Sperm Storage in a Liquid State (Variant I)

Sperm collected from the cauda epididymis of each animal were stored in a liquid state. After standard evaluation, each sperm sample was diluted in Salomon’s extender [4] (to a concentration of 100 × 10^6^ spermatozoa/mL). The samples were then incubated at room temperature for two hours and stored for up to six days in a refrigerator at 5 °C. 

### 2.3. Storage of Spermatozoa in the Epididymis (Variant II)

The second testicle with the epididymis was stored in scrotal sacs in the refrigerator for up to six days. In this variant, testicles with epididymides were randomly divided into three groups that were stored in the refrigerator for two days (D2, *n* = 11), four days (D4, *n* = 11), and six days (D6, *n* = 10). After refrigeration, sperm samples were collected, subjected to a standard evaluation, and diluted in Salomon’s extender (100 × 10^6^ spermatozoa/mL). 

### 2.4. Sperm Analysis

Sperm motility, viability, morphology, antioxidant status, and lipid peroxidation were determined after dilution of sperm samples (D0) and on subsequent days of storage (D2, D4, and D6).

#### 2.4.1. Assessment of Sperm Motility

Before the analysis, sperm samples were diluted with Dulbecco’s phosphate-buffered saline (DPBS; Gibco, Grand Island, NY, USA) in a 1:4 ratio (to obtain a concentration of 20–30 × 10^6^ spermatozoa/mL). The prepared samples were heated for approximately 5 min at 37 °C in a thermoblock (Thermo Block TDR-120, Göttingen, Germany). An aliquot (5 µL) of each sample was placed in a Makler counting chamber (Sefi-Medical Instruments Ltd., Haifa, Israel) preheated to 37 °C. Every sample was assessed in the CASA system and the Hamilton Thorne IVOS v. 12.3 sperm analyzer (Hamilton Thorne Bioscience, Beverley, MA, USA).

The evaluation involved software settings that were recommended by the manufacturer for analyses of gazelle/deer sperm: frame acquired—60, frame rate—60 Hz, minimum cell contrast—60, minimum cell size—5 pixels, straightness threshold—80%, low VAP cut-off—21.9 µm/s, low VSL cut-off—6 µm/s. Total motility (TMOT, %) and progressive motility (PMOT, %) were defined according to Hamilton Thorne requirements (VAP > 75 µm/s, STR > 80%).

#### 2.4.2. Sperm Viability Analysis

Sperm viability was assessed using the Muse^®^ Cell Analyzer (Luminex Corporation, Austin, TX, USA). Sperm samples were prepared for the analysis with the use of the Muse Count & Viability Kit (Luminex Corporation, Austin, TX, USA), according to the manufacturer’s recommendations. The reagent contains two dyes. The first dye penetrates the cell membrane and stains DNA, and nucleated cells are separated from non-nucleated cells and impurities. The second dye contains 7-aminoactinomycin D (7-AAD), which stains only cells with damaged cell membranes. This combination of dyes is used to differentiate between populations of live and dead cells. Before analysis, each sperm sample was diluted with DPBS to a concentration of 1 × 10^6^–1 × 10^7^ cells/mL. An aliquot of 20 µL was combined with 380 µL of the Muse Count & Viability Kit reagent and incubated for 5 min at room temperature. Sperm viability was determined with the use of the Muse^®^ Cell Analyzer.

#### 2.4.3. Assessment of Sperm Morphology

**Figure 1:**
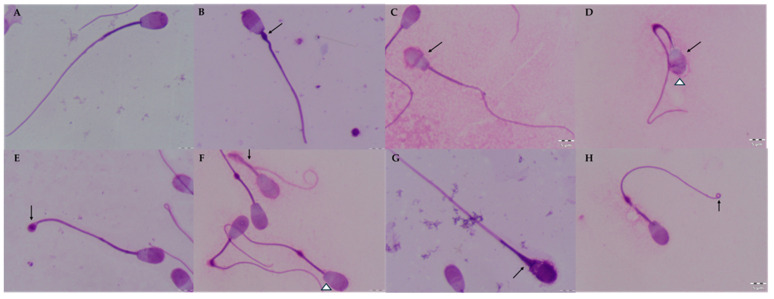
Morphological defects in European red deer epididymal spermatozoa stored in a liquid state and in the epididymides at 5 °C. (**A**)—normal spermatozoa; (**B**)—spermatozoa with proximal cytoplasmic droplets (⭡); (**C**)—spermatozoa with head defects (⭡); (**D**)—spermatozoa with head defects (⭡) and damaged acrosomes (Δ); (**E**)—spermatozoa with distal cytoplasmic droplets (⭡); (**F**)—spermatozoa with curled tails (⭡), spermatozoa with head defects (Δ); (**G**)—spermatozoa with midpiece defects (⭡); (**H**)—spermatozoa with a single bent tail (⭡). Scale bars = 5 µm.

The morphology of epididymal sperm was assessed by staining smears with the Giemsa method [27]. To prepare smear slides, 10 µL of the sperm sample (previously diluted with DPBS solution in a 1:4 ratio) was applied to a glass slide, spread over the entire slide, and left to dry at room temperature. Dried smears were fixed in a formalin buffer for 15 min, rinsed with water, and allowed to dry. The smears were then stained with Giemsa’s solution for 180 min. Smear slides were rinsed with distilled water and dried. In each sample, 200 sperm cells were counted under a phase contrast microscope at ×1000 magnification with oil immersion. During the analysis, normal spermatozoa; spermatozoa with intact acrosomes; spermatozoa with head, tail, and midpiece defects; and spermatozoa with proximal and distal cytoplasmic droplets were counted (Figure 1).

### 2.5. Activity of Antioxidant Enzymes and Lipid Peroxidation

On designated days, 0.5 mL aliquots of diluted sperm samples were collected in separate Eppendorf tubes. Each sample was diluted with 0.5 mL of 0.85% NaCl to a concentration of 50 × 10^6^ spermatozoa/mL. The samples were rinsed twice with 0.85% NaCl (3000× *g*, 5 min, 10 °C). The supernatant was discarded, and the precipitate was suspended in 1 mL of 0.85% NaCl. The prepared samples were frozen at −80 °C and stored for further analyses.

Sperm sediments were homogenized in a FastPrep^®^-24 apparatus (MP Biomedicals, Santa Ana, CA, USA) before the determination of antioxidant enzyme activity and MDA content. To obtain a clear supernatant, the samples were centrifuged at 12,000× *g* for 15 min at 10 °C, and the supernatant was transferred to clean Eppendorf tubes and stored at −80 °C for analysis.

#### 2.5.1. Superoxide Dismutase Activity

Superoxide dismutase activity was assessed with a Beckman Coulter DU 800 spectrophotometer (Beckman Coulter INC., Fullerton, CA, USA). The samples were prepared for analysis using the Ransod kit (Randox Laboratories, Crumlin, UK), according to the instructions provided by the manufacturer. SOD activity was measured at a wavelength of 505 nm. One unit (U) of SOD was defined as the amount of the enzyme that caused 50% inhibition of 2-(4-iodophenyl)-3-(4-nitrophenol)-5 phenyltetrazolium chloride (INT) reduction at 37 °C and pH 7.0. The results were expressed as U/10^6^ spermatozoa (spz).

#### 2.5.2. Glutathione Peroxidase Activity

Glutathione peroxidase activity was measured spectrophotometrically using a commercial Ransel assay (Randox Laboratories, Crumlin, UK) according to the manufacturer’s instructions. GPx activity was measured at a wavelength of 340 nm. One GPx unit was defined as the amount of the enzyme that catalyzes the oxidation of 1 µM of NADPH per minute at 37 °C and pH 7.2. The results were expressed as U/10^6^ spz.

#### 2.5.3. Catalase Activity

Catalase activity was measured using a commercial Catalase Assay Kit (Sigma-Aldrich Co., Saint Louis, MI, USA) according to the manufacturer’s instructions. Catalase activity was measured by determining the amount of H_2_O_2_ remaining after the reaction catalyzed by CAT. CAT activity was measured spectrophotometrically at 520 nm. One CAT unit was defined as the amount of the enzyme that decomposed 1 µM of H_2_O_2_ per minute at 25 °C and pH 7.0. The results were expressed as U/10^6^ spz.

#### 2.5.4. Malondialdehyde (MDA) Content

Lipid peroxidation in sperm cell membranes was evaluated by spectrophotometric determination of MDA content with a commercial BIOXYTECH^®^ MDA-586TM Assay Kit (OxisResearch, Burlingame, CA, USA) according to the manufacturer’s instructions. The measurement was performed at a wavelength of 586 nm. The results were expressed as μM MDA/10^6^ spz. 

### 2.6. Statistical Analysis

Data were processed statistically in Statistica v. 12.5 (StatSoft Incorporation, Tulsa OK, USA). The normality of data distribution was checked using the Shapiro–Wilk test. The results were analyzed with the use of non-parametric tests because the number of samples (*n*) differed across groups, and some variables did not have a normal distribution. Two independent samples were compared using the Mann–Whitney U test to determine the presence of significant differences between storage times in each variant and between storage variants on D2, D4, and D6. The results were presented as means ± SEM. 

In addition, the presence of significant correlations (*p* ≤ 0.05, *p* ≤ 0.01, *p* ≤ 0.001) between variables in each storage variant and storage time was determined by calculating Spearman’s rank correlation coefficient. The results of the correlation analysis were presented for the sixth day of storage (Supplementary Materials).

## 3. Results

### 3.1. The Effect of Storage Time on the Motility, Viability, Morphology, and Antioxidant Status of Spermatozoa Stored in a Liquid State and in the Epididymides

**Figure 2:**
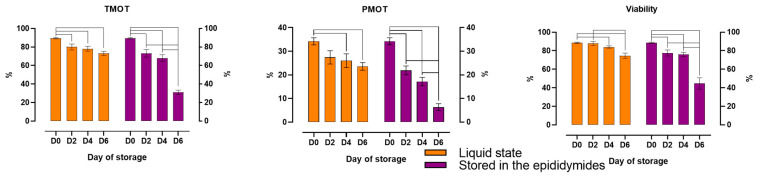
The effect of storage time on the motility and viability of spermatozoa stored in a liquid state and in the epididymides at 5 °C. TMOT, total motility; PMOT, progressive motility; Viability, live spermatozoa. The mean (±SEM) values of stored epididymal spermatozoa are presented. The results are significant at *p* ≤ 0.05.

Storage time significantly influenced the motility and viability of spermatozoa stored in a liquid state (variant I) and in the epididymides (variant II) (Figure 2). The percentage of motile sperm (TMOT) decreased significantly in both variants already on the second day of storage. The percentage of progressively motile spermatozoa (PMOT) and the percentage of spermatozoa with integral plasma membranes (Viability) also decreased significantly on D2 in sperm stored in the epididymides. In sperm stored in a liquid state, a significant decrease in PMOT and Viability was observed only on D4. However, on D6, TMOT and Viability exceeded 70%, and PMOT approximated 24% in variant I, whereas in variant II, TMOT and Viability decreased to approximately 30–40%, and PMOT decreased below 10%.

**Figure 3:**
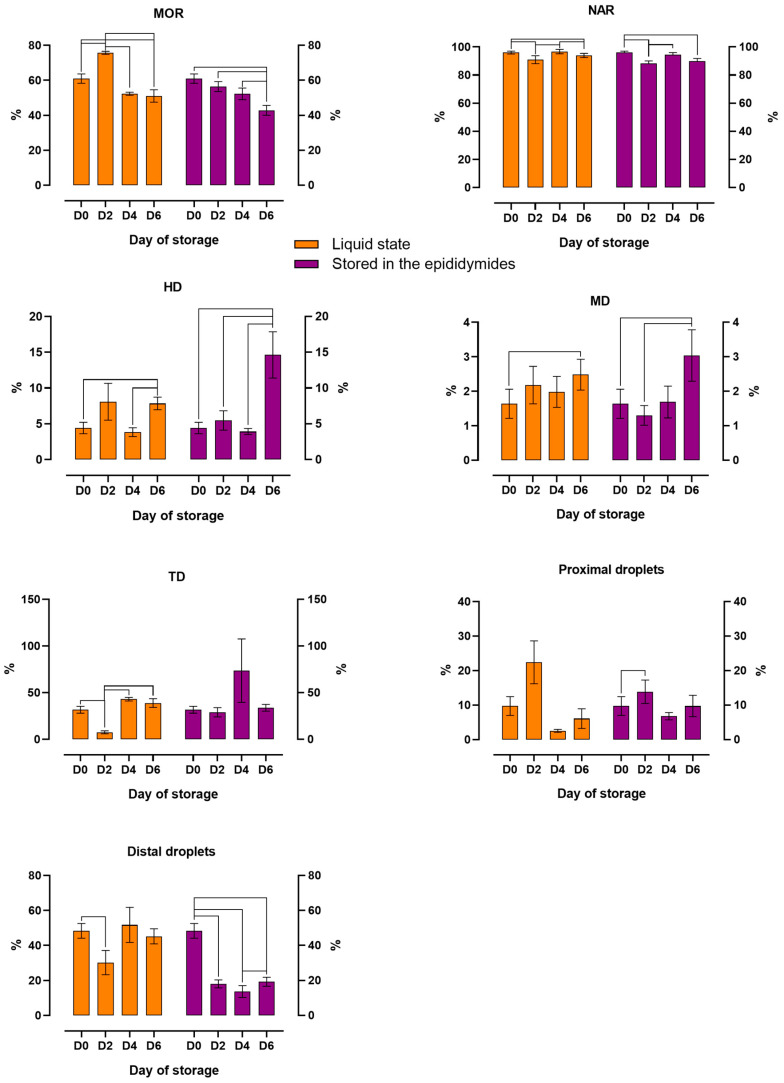
The effect of storage time on the morphology of spermatozoa stored in a liquid state and in the epididymides at 5 °C. MOR, normal sperm; NAR, normal apical ridge acrosomes; HD, head defects; MD, midpiece defects; TD, tail defects; proximal droplets; distal droplets. The mean (±SEM) values of stored epididymal sperm are presented. The results are significant at *p* ≤ 0.05.

The changes in the morphology of spermatozoa stored in a liquid state and in the epididymides for six days are presented in Figure 3. The percentage of spermatozoa with normal morphology (MOR) was highest on D2 in samples stored in a liquid state. The values of MOR decreased gradually over time in both variants. In variant I, significant differences in MOR values were noted between D0 and D2, and between D0 and D2 vs. D4 and D6. In variant II, MOR decreased significantly to around 45% only on D6, compared with around 55% in variant I. Significant changes in acrosome integrity were also observed during storage. In both storage variants, the percentage of spermatozoa with normal apical ridge acrosomes (NAR) was lowest on D2 and D6 relative to D0. The percentage of spermatozoa with head (HD), midpiece (MD), and tail defects (TD) increased over time. In both variants, significant differences (*p* ≤ 0.05) in HD and MD were noted between D0 and D6. A significant increase (*p* ≤ 0.05) in HD and MD was observed on D6 in spermatozoa stored in the epididymides relative to those stored in a liquid state. The percentage of spermatozoa with tail defects (TD) also increased over time in both variants. However, significant differences (*p* ≤ 0.05) in TD were noted only in variant I between D0 and D2, and between D2 vs. D4 and D6. The percentage of spermatozoa with proximal cytoplasmic droplets was highest on D2 in both variants, but significant differences (*p* ≤ 0.05) in this parameter were observed only in variant II between D0 and D2. The percentage of spermatozoa with distal cytoplasmic droplets also decreased over time, in particular in variant II, where this parameter decreased from around 48% on D0 to around 20% on D6. In both variants, significant differences (*p* ≤ 0.05) in this parameter were observed between D0 and D2.

**Figure 4:**
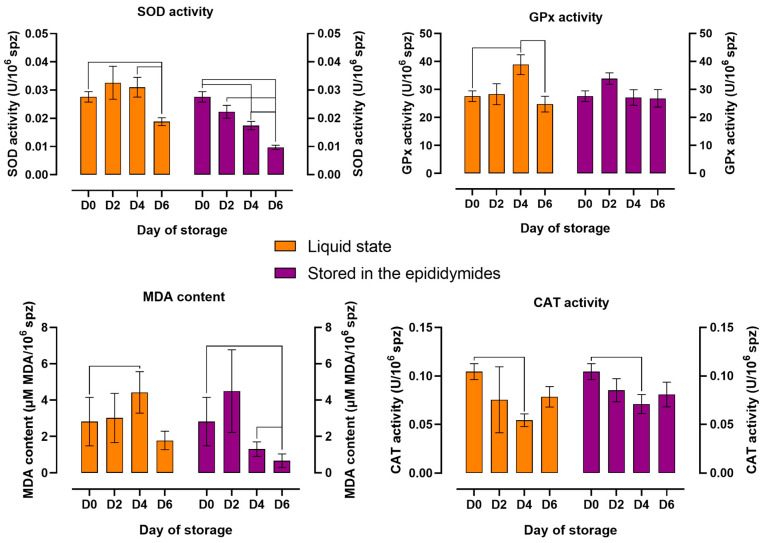
The effect of storage time on the activity of antioxidant enzymes and lipid peroxidation in spermatozoa stored in a liquid state and in the epididymides at 5 °C. SOD activity, superoxide dismutase activity; GPx activity, glutathione peroxidase activity; MDA content, malondialdehyde content; CAT activity, catalase activity. The mean (±SEM) values of stored epididymal sperm are presented. The results are significant at *p* ≤ 0.05.

The changes in the activity of antioxidant enzymes and lipid peroxidation in spermatozoa stored in a liquid state and in the epididymides for six days are presented in Figure 4. Significant changes in SOD activity were observed over time, subject to storage variant. In samples stored in a liquid state, SOD activity was highest on D2 and D4. In spermatozoa stored in the epididymides, SOD activity was highest on D0 and decreased on successive days of storage. Significant differences (*p* ≤ 0.05) in SOD activity were noted between D0 and D6 in both variants. In variant I, GPx activity and MDA content were highest on D4 and lowest on D6. Significant differences (*p* ≤ 0.05) in the values of both parameters were observed between D0 and D4 in variant I. In variant II, GPx activity and MDA content were highest on D2 and lowest on D6. Significant differences (*p* ≤ 0.05) in MDA content were noted between D0 and D6 in variant II. CAT activity was lowest on D4 in both variants, and significant differences (*p* ≤ 0.05) in this parameter were observed only between D0 and D4.

### 3.2. The Effect of Storage Variant on Sperm Motility, Viability, Morphology, and Antioxidant Status

The storage variant had a significant influence on the analyzed variables, depending on storage time (Table 1). The earliest significant differences between the compared variants were noted on D2 in MOR values, which can be attributed to higher TD values in variant II than in variant I. On D4, a significant decrease (*p* ≤ 0.05) in PMOT, Viability, Distal Droplets, SOD and GPx activity, and MDA content, and a significant increase (*p* ≤ 0.05) in Proximal Droplets were noted in epididymal sperm relative to sperm stored in a liquid state. On the last day of storage (D6), a further significant decrease in PMOT, Viability, Distal Droplets, and SOD activity, a decrease in TMOT values, and an increase in HD values were noted in variant II relative to variant I.

## 4. Discussion

This is the first study to analyze the influence of storage time and short-term storage method on changes in the morphology and antioxidant status of European red deer epididymal sperm. Previous research was conducted on other animal species [18,28,29,30], whereas very few studies examined cervids and reported only on morphological changes [31,32].

Sperm motility and viability are crucial for successful fertilization, and these parameters were also analyzed in the present study. Motility is a key determinant of sperm quality [33] and suitability for assisted reproductive technologies (ART). The integrity of the acrosomal membrane is essential for sperm functions, including motility [34]. In the current study, the motility of spermatozoa stored in a liquid state and in the epididymides was similar to that noted by other researchers [3,6,10,13,35]. The motility and viability of epididymal spermatozoa stored in a liquid state were maintained at a satisfactory level (above 70%) until the sixth day of storage, which could indicate that their functional capacity was retained. This finding corroborates the results of our previous study, which demonstrated that epididymal sperm stored in a liquid state for up to 10 days remained useful for reproductive purposes [6]. In spermatozoa stored in the epididymides, comparable levels of motility and viability were observed only until the second day of storage, and a significant decrease in these parameters (to approx. 31% and 44%, respectively) was noted on the sixth day of storage. Motility and viability are closely correlated parameters [5], and this observation was confirmed in this study in both storage variants (Tables S1 and S2, Figure S1). 

Storage induces many age-related changes that disrupt metabolic processes, increase lipid peroxidation, and cause damage to sperm structures [14,28,36,37]. In the present study, the percentage of spermatozoa with normal morphology decreased over time, but the prevalence of morphological defects differed across storage variants. The percentage of sperm with tail defects and, on the last day of storage (D6), with midpiece and head defects was significantly higher in spermatozoa stored in the epididymides than in a liquid state. These changes can undermine sperm motility and viability [34], which was observed on D2 in spermatozoa stored in the epididymides, but only on D4 in sperm stored in a liquid state. Interestingly, a greater decrease in the percentage of spermatozoa with cytoplasmic droplets (in particular distal droplets) was noted in sperm stored in the epididymides than in a liquid state. Cytoplasmic droplets are characteristic of immature sperm cells, and they are commonly found in epididymal sperm [38,39,40]. Cytoplasmic droplets are shed during maturation, and the results of the present study indicate that epididymal storage supported sperm maturation. During liquid storage, the decrease in the percentage of spermatozoa with cytoplasmic droplets was correlated with changes in motility, and similar observations had been previously made in the epididymal sperm of mice [41], which suggests that cytoplasmic droplets play an important role in motility regulation [38]. Other researchers found that cytoplasmic droplets are transient organelles that provide the necessary energy for the maturation of epididymal spermatozoa [25,41]. 

Disruptions in metabolic processes, such as lipid peroxidation, induce irreversible damage to sperm structures [14]. Stored spermatozoa become more susceptible to oxidative damage caused by ROS. Reactive oxygen species are produced in the mitochondria and plasma membranes of spermatozoa, and their generation is controlled by the antioxidant system composed of enzymatic and non-enzymatic antioxidants [42,43]. The enzymatic system of many animal species is composed mainly of GPx, CAT, and SOD [14,22,42]. SOD is considered the key enzyme in the antioxidant defense system of spermatozoa [21,44]. 

In the current study, SOD activity in spermatozoa stored in a liquid state decreased over time, and similar results were reported in the spermatozoa of rams [45], cattle, and buffalo bulls during liquid storage [18]. In sperm stored in the epididymides, a significant decrease in SOD activity was reported much earlier (on D2). In addition, on D4, SOD activity was higher in spermatozoa stored in a liquid state than in the epididymides, which could suggest that the antioxidant capacity of sperm cells is more effectively preserved during liquid storage. Interestingly, GPx activity in the epididymal storage variant remained fairly constant during the entire storage period, whereas in sperm cells stored in a liquid state, the GPx activity was highest on D4. In contrast, GPx activity in cattle and buffalo bull spermatozoa continued to decrease on successive days of storage [18]. 

The content of MDA, a common biomarker of lipid peroxidation, increased during storage. This observation suggests that peroxidation occurred much earlier during epididymal storage (MDA content peaked on D2) than liquid storage (D4). The increase in MDA content was accompanied by an increase in GPx activity. The activity of this antioxidant enzyme probably increased to counteract excessive ROS production [14,46,47]. 

Lipid peroxidation is triggered by ROS, and ROS generation is controlled by SOD, GPx, and CAT. SOD catalyzes the dismutation of superoxide radicals (O^2-^) to O_2_ and H_2_O_2_ [22,48,49]. The generated H_2_O_2_ is decomposed by CAT to oxygen and water. In addition, H_2_O_2_ is neutralized by GPx and glutathione reductase [22,49,50]. 

In the present study, CAT activity was low in both storage variants, which could suggest that H_2_O_2_ in deer spermatozoa is reduced mainly by GPx. This observation corroborates the previous findings of Drevet et. al. [20]. Similarly to other antioxidant enzymes, CAT activity decreased over time. Low CAT activity could also suggest that deer sperm contain trace amounts of this enzyme. However, further research involving more sensitive tests and molecular techniques is needed to validate this hypothesis since CAT was not detected in the spermatozoa of many animal species, including cattle, dogs, and boars [22,51,52]. Several authors reported extremely low levels of CAT activity in human and rat spermatozoa [53,54]. In turn, CAT was detected in stallion sperm [29]. 

The antioxidant status of deer spermatozoa has not been investigated to date, and the present study provides novel information that could be used to develop new methods for storing epididymal spermatozoa.

## 5. Conclusions

The study demonstrated that the storage time of up to 6 days significantly affects the quality of the epididymal spermatozoa of European red deer stored in a liquid state and in the epididymides. However, the motility, viability, morphology, and antioxidant status of deer spermatozoa are more effectively preserved during liquid storage than epididymal storage. Lipid peroxidation, morphological defects, and a decrease in SOD activity occurred earlier in spermatozoa stored in the epididymides than in a liquid state, which exerted a negative effect on sperm viability and motility. Low CAT activity in epididymal spermatozoa suggests that hydrogen peroxide in sperm cells is reduced mainly by GPx. Based on the present findings, liquid storage is more recommended for the short-term preservation of deer spermatozoa than epididymal storage. The results of this study also indicate that sperm stored in the epididymides at 5 °C for up to 4 days could be suitable for reproduction. Further comparative studies involving in vitro fertilization or artificial insemination are recommended to confirm these findings.

## Figures and Tables

**Table 1:** The effect of storage variant (liquid state vs. storage in the epididymides) on the motility, viability, morphology, and antioxidant status of European red deer spermatozoa stored at 5 °C.

Sperm Quality Parameters	Day of Storage					
	D2 *n* = 11		D4 *n* = 11		D6 *n* = 10	
	Variant I	Variant II	Variant I	Variant II	Variant I	Variant II
TMOT	80.00 ± 3.16	73.17 ± 4.11	78.14 ± 2.66	68.00 ± 3.66	73.00 ± 2.26 ^a^	31.00 ± 5.37 ^b^
PMOT	27.40 ± 2.79	21.92 ± 1.86	26.00 ± 2.90 ^a^	17.17 ± 1.77 ^b^	23.58 ± 1.59 ^a^	6.30 ± 1.48 ^b^
Viability	87.80 ± 22.23	77.56 ± 3.26	84.02 ± 1.27 ^a^	76.05 ± 2.37 ^b^	74.61 ± 2.84 ^a^	44.69 ± 6.22 ^b^
MOR	75.74 ± 0.88 ^a^	56.47 ± 2.86 ^b^	52.35 ± 0.89	52.27 ± 3.34	51.08 ± 3.58	42.85 ± 2.84
NAR	90.94 ± 2.83	88.21 ± 1.86	96.73 ± 1.56	94.54 ± 1.48	93.93 ± 1.51	89.75 ± 2.09
HD	8.08 ± 2.58	5.47 ± 1.35	42.97 ± 1.98	3.92 ± 0.42	7.85 ± 0.88 ^a^	14.63 ± 3.23 ^b^
MD	2.18 ± 0.55	1.30 ± 0.29	1.98 ± 0.45	1.69 ± 0.46	2.48 ± 0.45	3.04 ± 0.74
TD	7.48 ± 1.65 ^a^	28.99 ± 5.03 ^b^	42.97 ± 1.98	73.60 ± 33.94	38.89 ± 4.62	33.80 ± 3.63
Prox Drop	22.41 ± 6.21	13.87 ± 3.40	2.55 ± 0.45 ^a^	6.78 ± 1.04 ^b^	6.11 ± 2.85	9.73 ± 3.09
Dist Drop	30.22 ± 6.91	18.05 ± 2.28	51.75 ± 10.03 ^a^	13.65 ± 3.39 ^b^	45.22 ± 4.32 ^a^	19.28 ± 2.55 ^b^
SOD activity	0.03 ± 0.01	0.02 ± 0.01	0.03 ± 0.01 ^a^	0.02 ± 0.01 ^b^	0.02 ± 0.01 ^a^	0.01 ± 0.01 ^b^
GPx activity	28.32 ± 3.75	33.88 ± 2.07	38.90 ± 3.54 ^a^	27.17 ± 2.78 ^b^	24.75 ± 2.80	26.85 ± 3.12
MDA content	3.02 ± 1.35	4.50 ± 2.28	4.43 ± 1.14 ^a^	1.32 ± 0.40 ^b^	1.79 ± 0.50	0.67 ± 0.37
CAT activity	0.08 ± 0.03	0.09 ± 0.01	0.05 ± 0.01	0.07 ± 0.01	0.08 ± 0.01	0.08 ± 0.01

TMOT, total motility (%); PMOT, progressive motility (%); Viability, live spermatozoa (%); MOR, normal sperm (%); NAR, normal apical ridge acrosomes (%); HD, head defects (%); MD, midpiece defects (%); TD, tail defects (%); Prox Drop, Proximal Droplets (%); Dist Drop, Distal Droplets (%); SOD activity, superoxide dismutase activity (U/10^6^ spz); GPx activity, glutathione peroxidase activity (U/10^6^ spz); MDA content, malondialdehyde content (μm MDA/10^6^ spz); CAT activity, catalase activity (U/10^6^ spz). The mean (±SEM) values of stored epididymal sperm are presented. Values marked with different letters (^a,b^) denote significant differences between storage variants on the same day of storage at *p* ≤ 0.05.

## Data Availability

The data presented in this study are available on request from the corresponding author.

## Supplementary Materials

The following supporting information can be downloaded at: https://www.mdpi.com/article/10.3390/ani14111653/s1, Figure S1: Correlation coefficients describing the relationships between the quality parameters of European red deer epididymal spermatozoa stored in a liquid state (A) and in the epididymides (B) at 5 °C for 6 days. Table S1: Correlation coefficients describing the relationships between the quality parameters of European red deer epididymal spermatozoa stored in a liquid state at 5 °C for 6 days. Table S2: Correlation coefficients describing the relationships between the quality parameters of European red deer spermatozoa stored in the epididymides at 5 °C for 6 days.
