# Supplementary material for: Changes in the Morphology and Antioxidant Status of European Red Deer Sperm Stored in the Epididymides and in a Liquid State
Source: Animals (Basel). 2024 May 31;14(11):1653. doi: 10.3390/ani14111653 (PMC11171189; doi:10.3390/ani14111653)
Supplement: Supplementary file 1 [file animals-14-01653-s001.zip › Table S1 and S2.docx]

**Table S1.** Correlation coefficients describing the relationships between the quality parameters of European red deer epididymal spermatozoa stored in a liquid state at 5 °C for 6 days.

|  | TMOT | PMOT | Viability | MOR | NAR | HD | MD | TD | Proximal droplets | Distal droplets | SOD activity | GPx activity | MDA content | CAT activity |
| --- | --- | --- | --- | --- | --- | --- | --- | --- | --- | --- | --- | --- | --- | --- |
| TMOT | 1 | 0.772** | 0.832*** | 0.695* | -0.200 | 0.172 | 0.298 | -0.512 | 0.325 | 0.098 | 0.418 | -0.414 | -0.123 | -0.298 |
| PMOT |  | 1 | 0.749** | 0.408 | 0.067 | 0.046 | 0.236 | -0.221 | 0.071 | 0.601* | 0.327 | -0.239 | 0.074 | -0.236 |
| Viability |  |  | 1 | 0.462 | 0.021 | 0.266 | 0.175 | -0.245 | 0.253 | 0.133 | 0.161 | -0.315 | -0.084 | -0.517 |
| MOR |  |  |  | 1 | -0.119 | 0.035 | 0.545 | -0.916*** | 0.756** | -0.266 | 0.168 | -0.112 | -0.580* | -0.028 |
| NAR |  |  |  |  | 1 | -0.657* | -0.315 | 0.273 | 0.179 | 0.434 | -0.245 | 0.063 | -0.189 | 0.140 |
| HD |  |  |  |  |  | 1 | 0.441 | -0.168 | -0.098 | -0.399 | 0.196 | -0.112 | 0.385 | -0.490 |
| MD |  |  |  |  |  |  | 1 | -0.748** | 0.534 | -0.287 | 0.580* | 0.343 | -0.063 | -0.280 |
| TD |  |  |  |  |  |  |  | 1 | -0.780** | 0.448 | -0.329 | -0.028 | 0.538 | 0.084 |
| Proximal droplets |  |  |  |  |  |  |  |  | 1 | -0.436 | 0.102 | -0.014 | -0.714** | -0.285 |
| Distal droplets |  |  |  |  |  |  |  |  |  | 1 | 0.049 | 0.021 | 0.287 | 0.280 |
| SOD activity |  |  |  |  |  |  |  |  |  |  | 1 | 0.140 | 0.364 | -0.189 |
| GPx activity |  |  |  |  |  |  |  |  |  |  |  | 1 | 0.035 | 0.399 |
| MDA content |  |  |  |  |  |  |  |  |  |  |  |  | 1 | -0.112 |
| CAT activity |  |  |  |  |  |  |  |  |  |  |  |  |  | 1 |

* Significant at *p* ≤ 0.05; ** Significant at *p* ≤ 0.01; *** Significant at *p* ≤ 0.001; TMOT, total motility (%); PMOT, progressive motility (%); Viability, live spermatozoa (%); MOR, normal sperm (%); NAR, normal apical ride acrosomes (%); HD, head defects (%); MD, midpiece defects (%); TD, tail defects (%); Proximal droplets (%); Distal droplets (%); SOD activity, superoxide dismutase activity (U/10^6^); GPx activity, glutathione peroxidase activity (U/10^6^); MDA content, malondialdehyde content (μm MDA/10^6^); CAT activity, catalase activity (U/10^6^).

**Table S2.** Correlation coefficients describing the relationships between the quality parameters of European red deer spermatozoa stored in the epididymides at 5 °C for 6 days.

|  | TMOT | PMOT | Viability | MOR | NAR | HD | MD | TD | Proximal droplets | Distal droplets | SOD activity | GPx activity | MDA content | CAT activity |
| --- | --- | --- | --- | --- | --- | --- | --- | --- | --- | --- | --- | --- | --- | --- |
| TMOT | 1 | 0.873*** | 0.942*** | 0.322 | 0.571 | -0.565 | -0.140 | 0.474 | -0.152 | -0.316 | 0.061 | -0.170 | -0.237 | -0.347 |
| PMOT |  | 1 | 0.821** | -0.056 | 0.352 | -0.438 | 0.049 | 0.463 | -0.309 | -0.358 | -0.130 | -0.136 | -0.562 | -0.025 |
| Viability |  |  | 1 | 0.152 | 0.612 | -0.539 | -0.212 | 0.697* | -0.382 | -0.503 | 0.176 | -0.115 | -0.333 | -0.491 |
| MOR |  |  |  | 1 | 0.418 | -0.539 | -0.212 | -0.152 | 0.636* | 0.212 | 0.018 | -0.394 | 0.721* | -0.345 |
| NAR |  |  |  |  | 1 | -0.503 | 0.030 | 0.382 | 0.127 | -0.333 | -0.358 | -0.285 | 0.079 | -0.491 |
| HD |  |  |  |  |  | 1 | 0.370 | -0.479 | -0.103 | 0.261 | -0.055 | 0.685* | -0.006 | 0.503 |
| MD |  |  |  |  |  |  | 1 | -0.321 | 0.200 | 0.418 | -0.721* | -0.042 | 0.006 | 0.467 |
| TD |  |  |  |  |  |  |  | 1 | -0.564 | -0.806** | 0.358 | 0.055 | -0.394 | -0.442 |
| Proximal droplets |  |  |  |  |  |  |  |  | 1 | 0.333 | -0.442 | -0.212 | 0.455 | 0.042 |
| Distal droplets |  |  |  |  |  |  |  |  |  | 1 | -0.188 | -0.285 | 0.515 | 0.333 |
| SOD activity |  |  |  |  |  |  |  |  |  |  | 1 | 0.285 | 0.079 | -0.345 |
| GPx activity |  |  |  |  |  |  |  |  |  |  |  | 1 | -0.115 | 0.394 |
| MDA content |  |  |  |  |  |  |  |  |  |  |  |  | 1 | -0.018 |
| CAT activity |  |  |  |  |  |  |  |  |  |  |  |  |  | 1 |

* Significant at *p* ≤ 0.05; ** Significant at *p* ≤ 0.01; *** Significant at *p* ≤ 0.001; TMOT, total motility (%); PMOT, progressive motility (%); Viability, live spermatozoa (%); MOR, normal sperm (%); NAR, normal apical ride acrosomes (%); HD, head defects (%); MD, midpiece defects (%); TD, tail defects (%); Proximal droplets (%); Distal droplets (%); SOD activity, superoxide dismutase activity (U/10^6^); GPx activity, glutathione peroxidase activity (U/10^6^); MDA content, malondialdehyde content (μm MDA/10^6^); CAT activity, catalase activity (U/10^6^).
