# Supplementary figures and images for: Changes in the Morphology and Antioxidant Status of European Red Deer Sperm Stored in the Epididymides and in a Liquid State
Source: Animals (Basel). 2024 May 31;14(11):1653. doi: 10.3390/ani14111653 (PMC11171189; doi:10.3390/ani14111653)

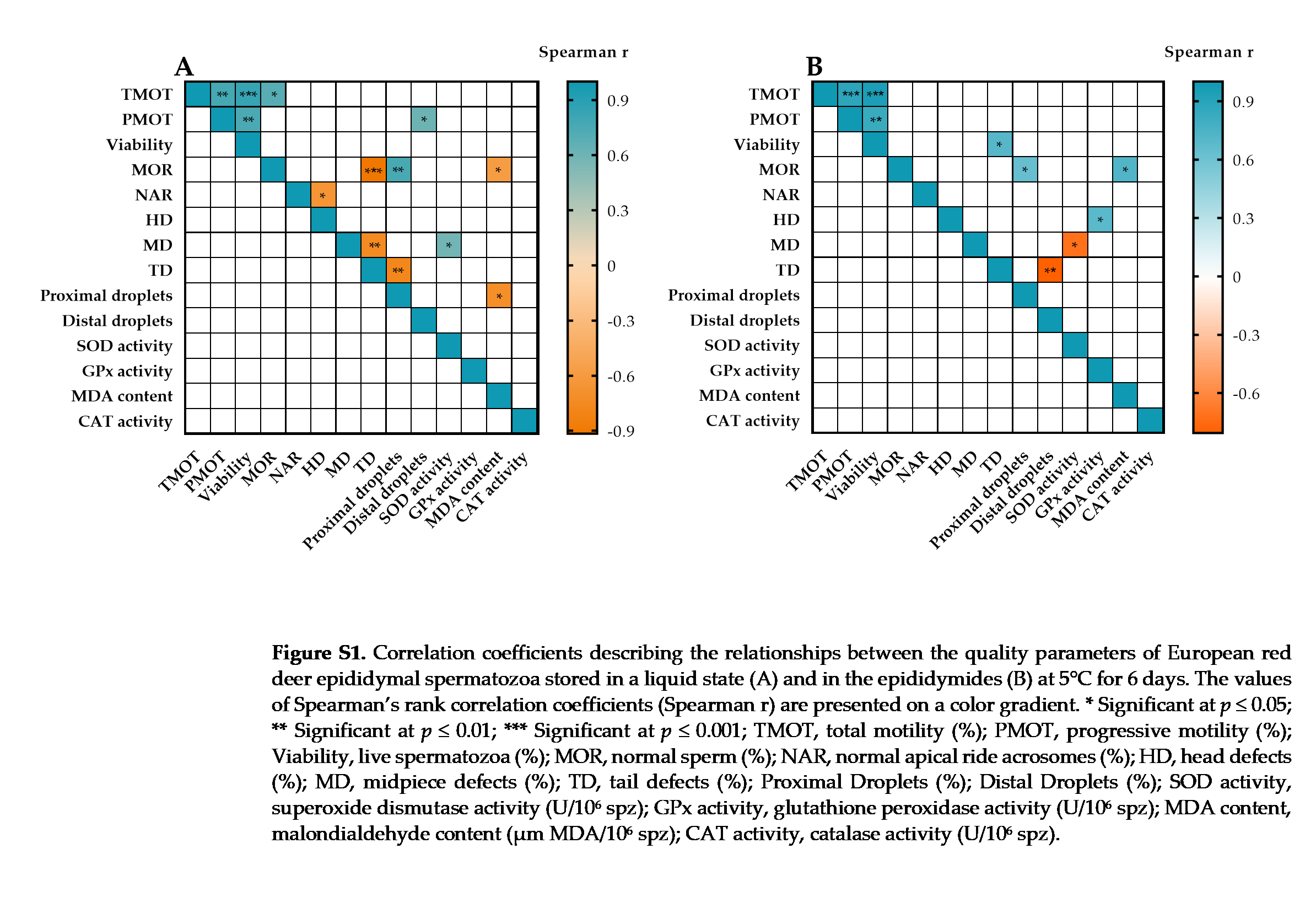

Supplement: Supplementary file 1 [file animals-14-01653-s001.zip › Suplementary Figure S1.tif]
